# Early immunohistochemical detection of pulmonary micrometastases in dogs with osteosarcoma

**DOI:** 10.1186/s13028-021-00608-9

**Published:** 2021-11-03

**Authors:** Mikael Kerboeuf, Erling Olaf Koppang, Anita Haug Haaland, Frode Lingaas, Øyvind Sverre Bruland, Jon Teige, Lars Moe

**Affiliations:** 1 Department of Companion Animal Clinical Sciences, Faculty of Veterinary Medicine, Norwegian University of Life Sciences, Elizabeth Stephansens vei 15, 1433 Ås, Norway; 2 Department of Preclinical Sciences and Pathology, Faculty of Veterinary Medicine, Norwegian University of Life Sciences, Elizabeth Stephansens vei 15, 1433 Ås, Norway; 3 Institute of Clinical Medicine &, Faculty of Medicine, Department of Oncology, The University of Oslo &, Norwegian Radium Hospital, Oslo University Hospital, Ullernchausseen 70, 0379 Oslo, Norway

**Keywords:** Bone cancer, Canine, Lung metastasis, Metastasis model, Pulmonary metastasis

## Abstract

**Background:**

Despite decades of research, the early phases of metastatic development are still not fully understood. Canine osteosarcoma (OS) is a highly aggressive cancer, with a high metastatic rate (> 90%), despite a low overt metastatic prevalence at initial diagnosis (< 15%). Canine OS is generally regarded as a good clinically relevant model for human OS. The aim of this hypothesis-generating study was to evaluate a method to detect pulmonary micrometastases and study their prevalence in dogs with OS without macroscopic metastases. We prospectively enrolled dogs with OS that received no cancer-specific treatment (n = 12) and control dogs without cancer (n = 2). Dogs were necropsied and sampled immediately after euthanasia. The OS dogs were classified as having macroscopic metastases (n = 2) or not (n = 10). We immunohistochemically stained one tissue sample from each of the seven lung lobes from each dog with a monoclonal antibody (TP-3) to identify micrometastases (defined as clusters of 5–50 tumour cells), microscopic metastases (> 50 tumour cells) and TP-3 positive single cells (< 5 tumour cells).

**Results:**

We showed that pulmonary micrometastases easily overseen on routine histology could be detected with TP-3. Pulmonary micrometastases and microscopic metastases were present in two dogs with OS without macroscopic metastases (20%). Micrometastases were visualised in three (43%) and four (57%) of seven samples from these two dogs, with a mean of 0.6 and 1.7 micrometastases per sample. Microscopic metastases were present in one (14%) and four (57%) of seven samples from the same two dogs, with a mean of 0.14 and 1.0 microscopic metastases per sample. There were four (57%) and two (29%) samples with neither microscopic metastases nor micrometastases for each of these two dogs. The prevalence of pulmonary micrometastases (20%) was significantly lower than expected (> 90%) based on commonly expected metastatic rates after amputation (P < 0.0001). There was no statistically significant difference in the number of TP-3 positive single cells in between groups (P = 0.85).

**Conclusions:**

Pulmonary micrometastases could be detected with TP-3 immunohistochemistry in a subset of dogs with OS before macroscopic metastases had developed. We propose that dogs with spontaneous OS represent clinically relevant models to study early micrometastatic disease.

## Background

Cancer is currently ranked as a major leading cause of death in humans and dogs, mainly due to metastatic disease [1–6]. Historically, murine cancer models have proven useful in understanding many of the underlying mechanisms of cancer, albeit with some limitations [7]. This is especially true when developing new therapeutics, as the majority have failed to reach the clinic. Micrometastases have been studied in some spontaneous canine cancer forms [8–12]. Their presence in lymph nodes, peripheral blood and bone marrow has been investigated in dogs with mammary carcinoma, as well as in lymph nodes of dogs with some other carcinomas and mast cell tumours. In humans, micrometastases have been studied in lymph nodes, bone marrow, lungs, liver, pleural or peritoneal cavities and peripheral blood in several forms of cancer [13–22].

Spontaneous canine osteosarcoma (OS) is considered a good model for human OS [23–30]. Most dogs with OS succumb to the disease, with the majority dying or being euthanized due to metastatic disease [31]. Although radiographically detectable metastases are uncommon at presentation (< 15–17%), most dogs eventually develop metastatic disease (> 90%) [31–35]. A question that remains unanswered is where the disseminated cells reside before macroscopic metastases develop. The main target organs for metastatic OS, both in humans and dogs, are the lungs and bones [29, 31, 34–37]. Bruland et al. found tumour cells in the bone marrow in 63% of human OS patients at presentation [14]. Amongst those presenting with overt metastases, the prevalence of tumour cells in the bone marrow was 92%. To our knowledge, micrometastases in the lungs have not been prospectively investigated either in human or canine OS.

The monoclonal antibody TP-3 binds selectively to a sarcoma-associated cell surface membrane antigen related to osteoblastic differentiation [38, 39]. The antigen is a monomeric polypeptide with alkaline phosphatase activity and a molecular weight of 80 kDa. TP-3 has been shown to bind to all evaluated OS cases in dogs [40, 41]. Similarly, various human sarcomas express the antigen, including all human OS cases examined [38, 39].

The aim of this hypothesis-generating study was to evaluate the use of TP-3 immunohistochemistry (IHC) on frozen tissue sections as a tool to detect pulmonary micrometastases and to study their prevalence in dogs with spontaneous OS before the development of macroscopic metastasis.

## Methods

### Study population

This study was conducted as a prospective case series of necropsied dogs with OS (OS+) and control dogs without OS (OS−). All cases were privately owned dogs presented to the Veterinary Teaching Hospital and private practices. Owners signed a participation consent form before the dogs were euthanized and necropsied. Dogs included in the OS + group could be of any breed, sex and age, had to have an appendicular primary tumour location and a confirmed histopathological OS diagnosis. Dogs that had undergone surgical treatment, except for diagnostic incisional biopsies, or other treatments except for pain-relieving drugs (opioids and NSAIDs), were excluded. For inclusion into the OS− group, dogs could be of any breed, sex and age, and had to have been euthanized for non-cancer-related disease. Hence, dogs with a previous history of cancer or a histopathological cancer diagnosis at necropsy were excluded from the OS− group.

### Necropsy and tissue collection

According to our protocol, all dogs had to be necropsied within two hours after euthanasia. Standard necropsy procedure was followed, with all organ systems being inspected macroscopically. Based on necropsy results, dogs in the OS + group were further classified as having macroscopic pulmonary metastases (OS + /Met +) or not (OS + /Met−). Tissue samples from all major organ systems (kidneys, lungs, liver, spleen, adrenal glands, myocardium, skeletal muscles and intestines) and any lesions suspected of being metastatic or neoplastic were collected and formalin-fixed. All formalin-fixed paraffin-embedded samples were stained with haematoxylin & eosin (H&E) and examined microscopically. Four tissue samples (sample size of approximately 1 × 1 × 1 cm) were taken from each of the seven lung lobes from each dog. Two were taken from the peripheral areas of the lobes, where only small bronchi were present, and two from the central, close to the main stem bronchi. Samples from all areas were collected for both formalin fixation and snap freezing in cold isopropanol (− 20 °C), quickly followed by submersion in liquid nitrogen and storage at − 80 °C. For dogs in the OS + /Met + group, samples for IHC analysis were taken from the same anatomical regions while avoiding macroscopic metastases. In addition, tissue samples from macroscopic metastases were formalin-fixed, stained with H&E and examined microscopically. Six of the seven samples from the peripheral areas and one of the seven from the central were picked at random, using an online random number generator (www.random.org) for further IHC analysis.

### IHC staining

IHC TP-3 staining was performed on frozen tissue sections. Snap frozen samples were sliced into 7 μm sections with a cryostat at − 25 °C. The tissue sections were mounted on poly-lysin-coated slides (Superfrost™ Plus, Thermo Fisher Scientific, Oslo, Norway) and dried at room temperature for one hour. The slides were then stored at − 80 °C until further preparation. IHC sections were labelled using the peroxidase-conjugated immune-polymer method (EnVision™, Dako, Glostrup, Denmark). The sections were first fixed in cold acetone (− 20 °C) for 10 min, followed by airdrying for 10 min. Endogenous peroxidase activity was inhibited by immersing the slides in a cold (4 °C) 0.3% H_2_O_2_ solution in phosphate-buffered saline (PBS) for 10 min. To prevent non-specific binding, the sections were blocked using a 1:50 solution of normal goat serum in 5% bovine serum albumin in tris-buffered saline (BSA/TBS) for 30 min. Sections were incubated with the purified IgG 2A monoclonal murine antibody (TP-3, 5 μg/mL, Norwegian Radiumhospital, Oslo, Norway), diluted in 1% BSA/TBS, for 60 min. The samples were then incubated with the secondary antibody (EnVision™, Dako, Glostrup, Denmark) for 30 min. Finally, immunolabelled tissues were developed using a 3-amino-9-ethylcarbazole (AEC) substrate chromogen (EnVision™, Dako, Glostrup, Denmark) incubated for 8 min, then counterstained with Mayer’s haematoxylin. The slides were mounted with coverslips using a water-soluble mounting medium (Aquatex®, Merck, Darmstadt, Germany) and left to dry at room temperature overnight. Negative controls were stained without primary antibodies. All washing steps of the IHC procedure were done by immersing the slides in three changes of PBS, each for 5 min at room temperature. All incubations were done at room temperature in a moisture chamber placed on a rotation table. A section containing both micrometastases and macroscopic metastases was used as a positive control for each staining.

### IHC analysis

Each slide was scanned for microscopic metastases, micrometastases and TP-3 positive single cells. Micrometastases were defined as clusters of ≥ 5 and ≤ 50 TP-3 positive cells. Clusters > 50 cells were defined as microscopic metastases, while clusters of < 5 TP-3 positive cells were defined as TP-3 positive single cells.

IHC stained slides were evaluated using a Zeiss AX10 microscope, equipped with a Zeiss axiocam 506 color camera, coupled with Zen pro 2012 (blue edition) image acquiring software (Carl Zeiss Microscopy GmbH, Jena, Germany). The total number of microscopic metastases and micrometastases, if present, were counted in the entire slide for each sample. The number of TP-3 positive single cells was counted in 10 high-power fields (HPF, defined as one field at 400x, equivalent to 0.196 mm^2^ for the microscope used). Areas with folded up tissue were excluded from the analysis. Slides where staining was too weak to identify positive cells or significant unspecific staining was present, were also excluded.

### Statistical analysis

All statistical analyses were performed using JMP pro 15.1.0 (SAS Institute Inc., Cary, NC). The mean number of TP-3 positive single cells per 10 HPF per lung lobe was compared between groups using Wilcoxon rank-sum tests for each pair and an unpaired Kruskal–Wallis test. The mean number of TP-3 positive single cells per 10 HPF for the different lung lobes (anatomical division) was compared between all the dogs combined and in between groups using Wilcoxon rank-sum tests for each pair and an unpaired Kruskal–Wallis test. The prevalence of micrometastases was tested against the expected prevalence (> 90%, based on the post-surgical metastatic rate) using a binomial test. P-values < 0.05 were considered statistically significant for statistical testing.

## Results

### Study population

**Fig. 1:**
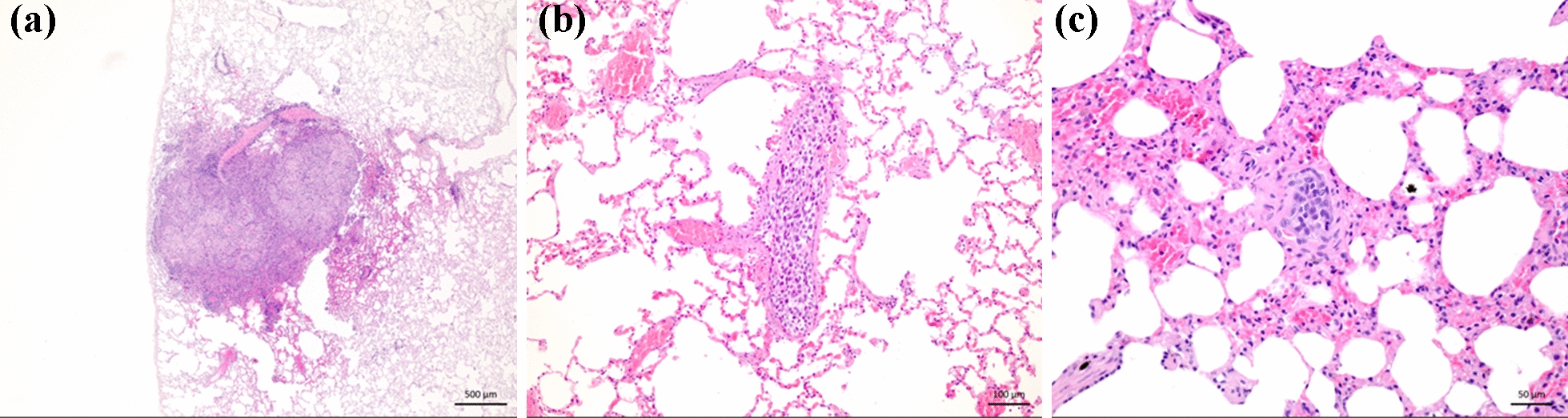
**a** Section of lung tissue from a dog with osteosarcoma with macroscopic metastases (case 2) showing a macroscopic metastasis (25 × magnification). Section of lung tissue from a dog with osteosarcoma without macroscopic metastases (case 5) showing **b** a microscopic metastasis lodged within a pulmonary arteriole (100 × magnification), and **c** a presumed micrometastasis within the alveolar septa (200 × magnification). Formalin-fixed paraffin-embedded tissue, haematoxylin & eosin stain

Cases were enrolled and necropsied between 2012 and 2020. In total 14 dogs were included in the study, ten in the OS + /Met- group, two in the OS + /Met + group and two in the OS−/Met− group (see Tables 1 and 2). OS + cases were confirmed based on clinical signs, diagnostic imaging, and histopathologic examination after H&E staining. The mean age was 5.6 years (median six years, range 1–11 years). There were eight (57%) male and six (43%) female dogs. The mean time from clinical presentation to euthanasia due to OS was 33 days (median 9.5 days, range 1–155 days). No neoplastic disease other than OS was found at necropsy in any of the dogs. The only significant pathological changes in the lungs were the macroscopic metastases seen in the two OS + /Met + dogs (Fig. 1a) and microscopic metastases (Fig. 1b) and suspected micrometastases (Fig. 1c) in some of the OS + /Met- dogs.

**Table 1:** Overview of the clinical characteristics, final diagnosis and tumour location for the dogs necropsied with primary appendicular osteosarcoma based on haematoxylin & eosin staining and controls included in the prospective study

Case	Breed	Sex	Age -years	Final diagnosis	Location of primary tumour
1	Mixed breed	M	1	Osteosarcoma	Left distal radius
2	Schnauzer	F	8	Osteosarcoma, osteoblastic	Right distal radius
3	Newfoundland dog	M	8	Osteosarcoma	Left distal radius
4	Siberian husky	M	3	Osteosarcoma, fibroblastic	Left proximal humerus
5	Irish wolfhound	F	6	Osteosarcoma	Right distal tibia
6	English setter	M	8	Osteosarcoma	Right distal ulna
7	Pointer	F	4	Osteosarcoma	Left distal tibia
8	Shar Pei	M	11	Osteosarcoma	Right proximal humerus
9	Rottweiler	F	9	Osteosarcoma, osteoblastic	Left proximal humerus
10	German shepherd	F	3	Osteosarcoma, osteoblastic	Left distal radius
11	Flat-coated retriever	M	3	Osteosarcoma	Left distal radius
12	Leonberger	M	6	Osteosarcoma	Left distal radius
13	Dalmatian	M	8	Urolithiasis	N.A
14	Shetland Sheepdog	F	1	Behavioural problems	N.A

*N.A* Not applicable

**Table 2:** Overview of the TP-3 immunohistochemical findings for the dogs in Table 1

Group	Case	Microscopic metastases	Micrometastases	Days from presentation to euthanasia
OS + /Met +	1	No	No	36
	2	No	No	28
OS + /Met−	3	No	No	1
	4	No	No	5
	5	Yes	Yes	10
	6	No	No	4
	7	No	No	32
	8	No	No	3
	9	Yes	Yes	155
	10	No	No	106
	11	No	No	9
	12	No	No	4
OS−/Met−	13	No	No	N.A
	14	No	No	N.A

The dogs were divided into three groups,—with macroscopic metastases (OS + /Met +),—without macroscopic metastases (OS + /Met−) and—without osteosarcoma (OS−/Met−). The presence (yes) or absence (no) of microscopic metastases (cluster of > 50 TP-3 positive cells) and micrometastases (cluster of 5–50 TP-3 positive cells) was recorded for each dog. (*N.A* Not applicable)

### Pulmonary micrometastases and microscopic metastases

**Fig. 2:**
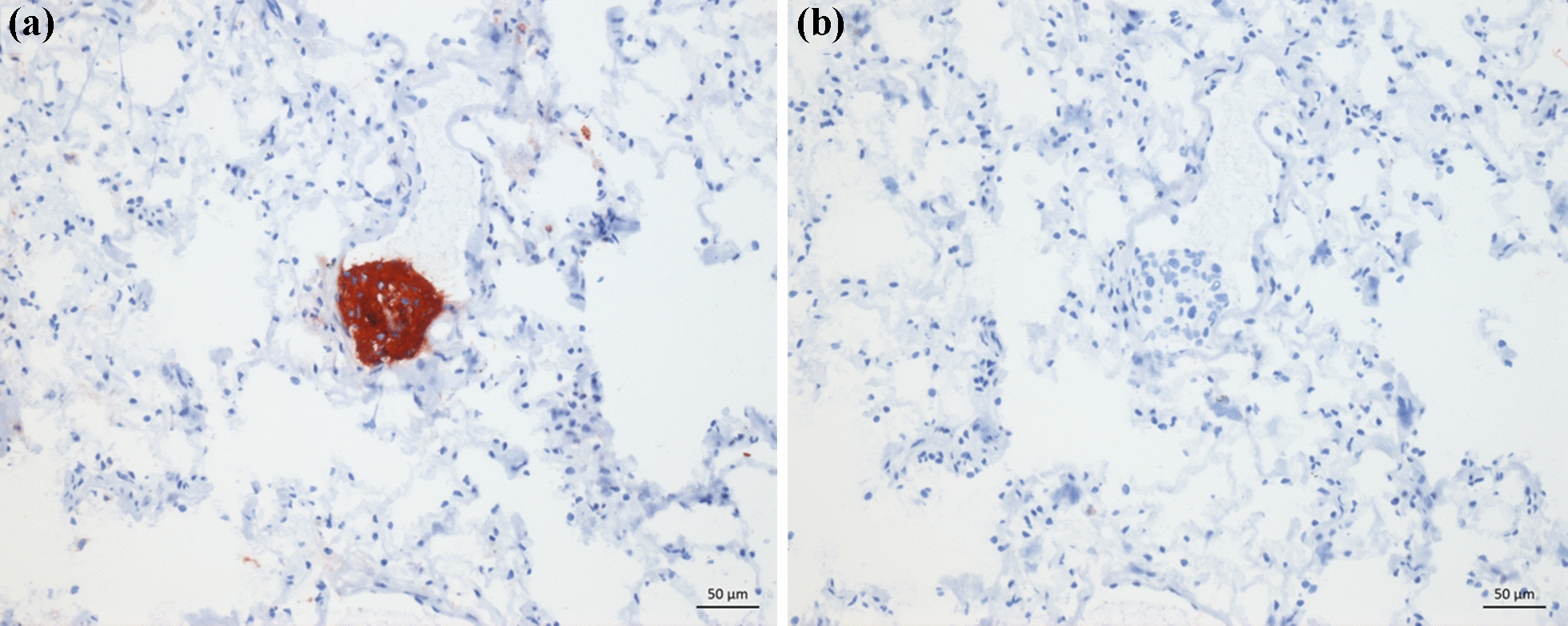
**a** Section of lung tissue from a dog with osteosarcoma without macroscopic metastases (case 9) showing immunolabeling (red staining) with tumour protein-3 (TP-3) of a pulmonary micrometastasis (defined as a cluster of 5–50 TP-3 positive cells). The micrometastasis is lodged within a pulmonary arteriole. **b** Section of the same tissue as in **a**, but without TP-3 antibody (negative control). Snap frozen tissue, immunoperoxidase stain, TP-3, AEC chromogen and haematoxylin counterstain, 200 × magnification

**Fig. 3:**
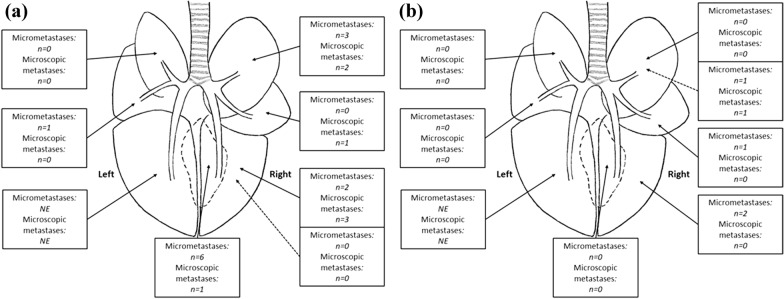
Distribution and number of micrometastases (cluster of 5–50 TP-3 positive cells) and microscopic metastases (cluster of > 50 TP-3 positive cells) in two dogs with osteosarcoma without macroscopic metastases. **a** represents case 5 and **b** represents case 9. Each arrow and box correspond to a specific lung lobe, from the right top side and clockwise: Right cranial lobe, right medial lobe, right caudal lobe, accessory lobe, left caudal lobe, left medial lobe, and left cranial lobe. The solid arrows indicate samples from the peripheral lung tissue, while the dotted arrows indicate samples from the central lung tissue (*NE* Not examined)

A total of 98 lung samples underwent IHC TP-3 staining and histological evaluation, in addition to the positive and negative controls. Tumour cells throughout all micrometastases and microscopic metastases showed a strong and seemingly cytoplasmatic TP-3 staining, as seen in Fig. 2. Depending on their size, micrometastases were either found lodged within pulmonary arterioles (Fig. 2) or the capillaries of the alveolar septa. In larger metastatic lesions, TP-3 staining varied more. Here, cells in the periphery showed strong staining, while those towards the centre stained only weakly or not at all. Micrometastases were present in two dogs (20%) in the OS + /Met− group (Table 2), whereas we found none in the ten remaining cases (eight in the OS + /Met− and both in the OS + /Met + group) or the OS−/Met− group. Microscopic metastases were present in these same two dogs (Table 2). Micrometastases were found in three (43%) and four (57%) of the seven samples examined for each of the two dogs. The total number of micrometastases in each dog was four and 12, respectively, with a mean of 0.6 (range 0–2) and 1.7 (range 0–6) micrometastases per sample. Microscopic metastases were found in one (14%) and four (57%) of the seven samples in the same two dogs. The total number of microscopic metastases was one and seven, respectively, with a mean of 0.14 (range 0–1) and 1.0 (range 0–3) microscopic metastases per sample. Figure 3 shows the distribution of metastases in each lung lobe. There were four (57%) and two (29%) samples for each of the two dogs with neither microscopic metastases nor micrometastases.

### TP-3 positive single cells

**Fig. 4:**
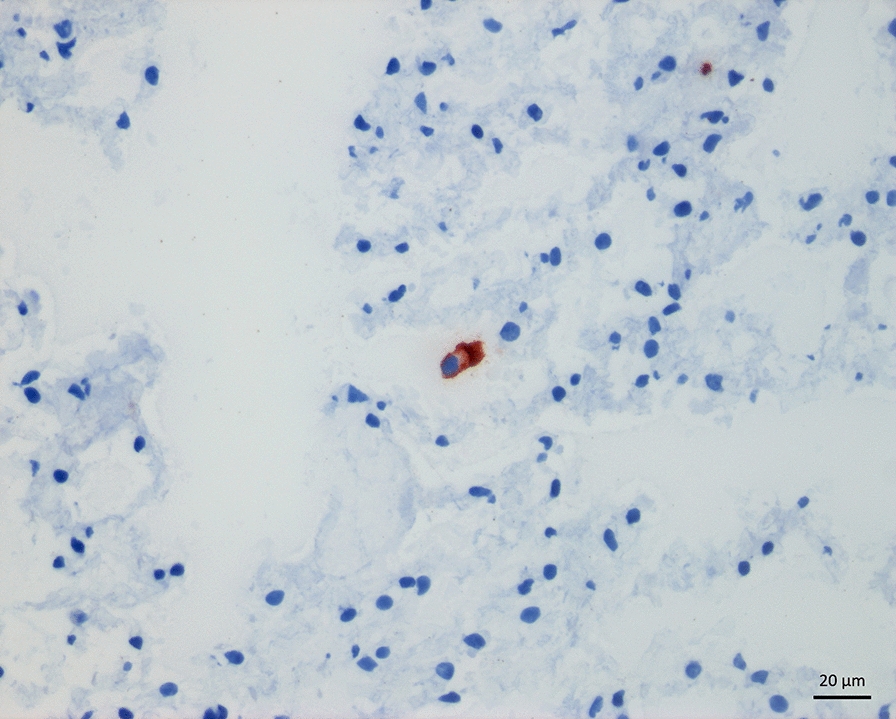
Section of lung tissue from a dog without neoplastic disease (case 14) showing immunolabeling (red staining) with tumour protein-3 (TP-3) of a single cell. Based on staining pattern and intensity, as well as cytoplasmatic and nuclear morphology, the cell represents a dislodged respiratory epithelial cell. Snap frozen tissue, immunoperoxidase stain, TP-3, AEC chromogen and haematoxylin counterstain, 400 × magnification

**Fig. 5:**
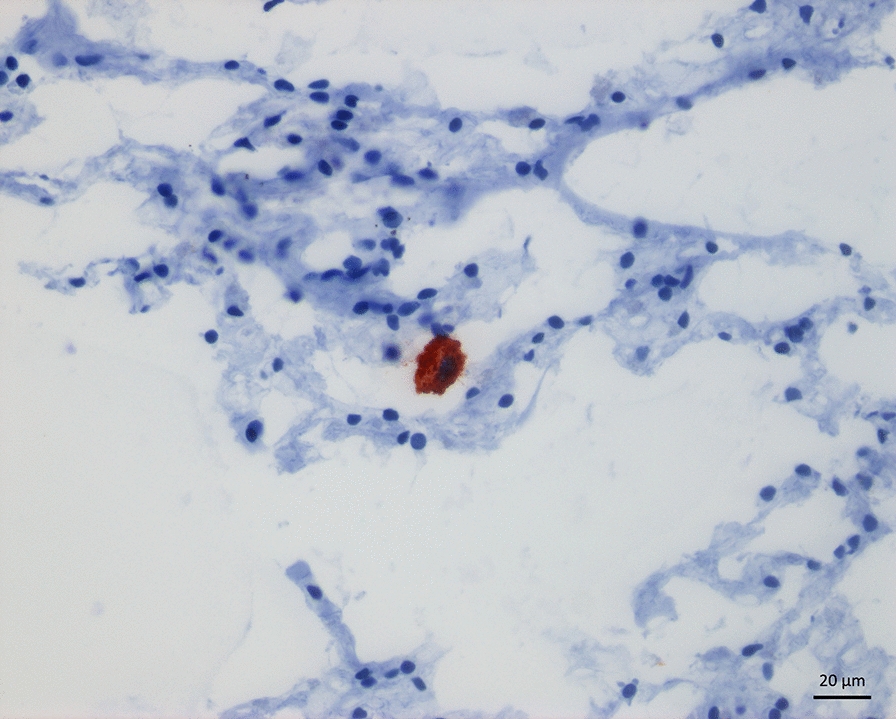
Section of lung tissue from a dog with osteosarcoma without macroscopic metastases (case 5) showing immunolabeling (red staining) with tumour protein-3 (TP-3) of a single cell. Based on staining pattern and intensity, as well as cytoplasmatic and nuclear morphology, this cell may represent a tumour cell. Snap frozen tissue, immunoperoxidase stain, TP-3, AEC chromogen and haematoxylin counterstain, 400 × magnification

TP-3 positive single cells were present in the lung parenchyma in all samples in all dogs. These were either scattered respiratory epithelial cells or metastatic OS cells. Most TP-3 positive single cells were identified as dislodged respiratory epithelium, based on cytoplasmatic morphology, staining pattern and nuclear characteristics *(*Fig. 4). They showed asymmetrical staining, which was stronger along the ciliated brush border and weaker on the opposite side of the cell. In addition, the majority had small to moderately sized eccentric nuclei and no visible nucleoli. In some cases, TP-3 positive single cells were more compatible with tumour cells, showing a strong and homogenous cytoplasmatic staining, with large nuclei and distinct nucleoli (Fig. 5). In most cases, we could not reliably distinguish the two cell populations, and as such, they were all counted as TP-3 positive single cells.

The mean number of TP-3 positive single cells per 10 HPF per sample was 6.5 (median 6.5, range 5.9–7) for the OS + /Met + group, 120.5 (median 15.2, range 3–941.9) for the OS + /Met- group and 17.9 (median 17.9, range 4.7–31.1) for the OS−/Met− group. There was no statistically significant difference in the mean number of TP-3 positive single cells per 10 HPF per sample between the groups (P = 0.85). There was no statistically significant difference between the means of the OS + /Met− group and the OS + /Met + (P = 0.75) or OS−/Met− (P = 0.75) group, nor between the OS + /Met + and OS−/Met− group (P = 1.0). There were no statistically significant differences in the mean number of TP-3 positive single cells per 10 HPF between any of the different lung lobes (anatomical division), neither when combining all the dogs (P = 0.96), nor within the different groups (for OS + /Met + , P = 0.82, OS + /Met−, P = 0.89 and OS−/Met−, P = 0.62).

### Normal structures stained with TP-3

**Fig. 6:**
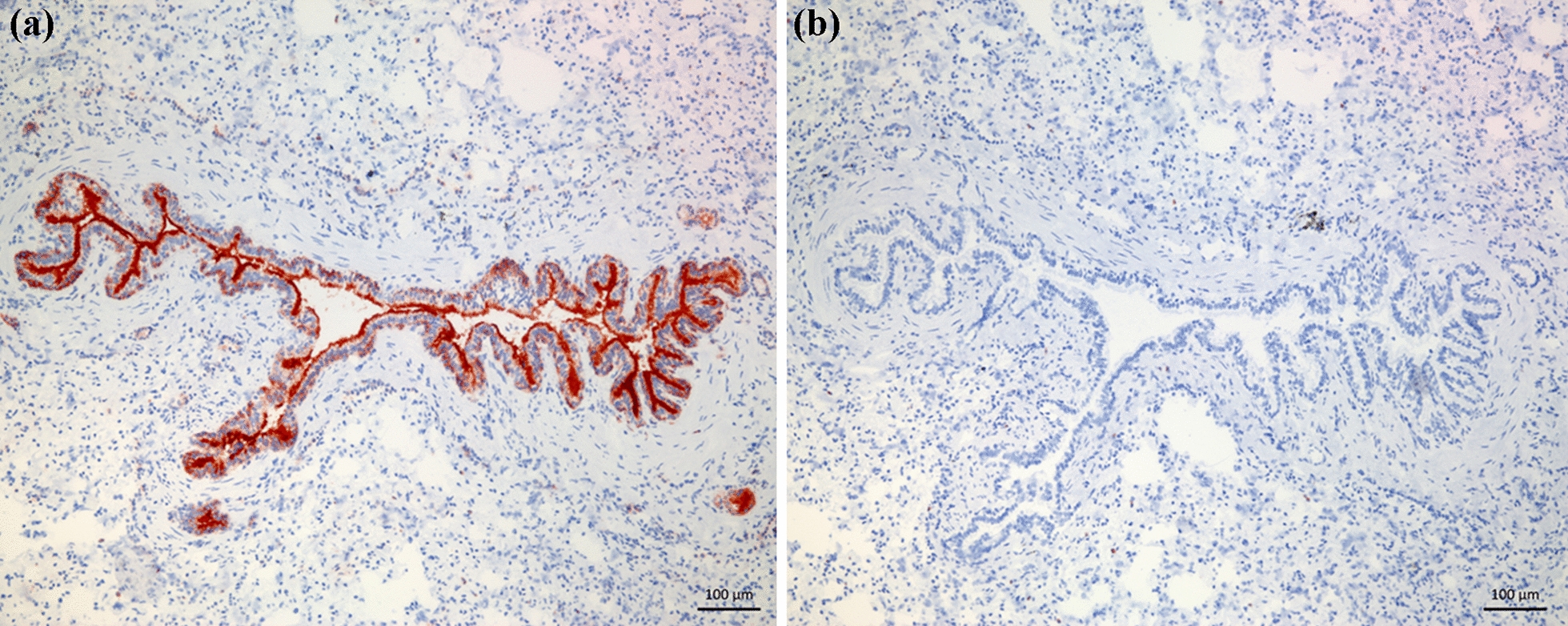
**a** Section of lung tissue from a dog (case 3) showing immunolabeling (red staining) with tumour protein-3 (TP-3) of the respiratory bronchiolar epithelium. An asymmetrical staining pattern can be seen, with a stronger reaction towards the ciliated brush border. **b** Section of the same tissue as in **a**, but without TP-3 antibody (negative control). Snap frozen tissue, immunoperoxidase stain, TP-3, AEC chromogen and haematoxylin counterstain, 100 × magnification

TP-3 staining was observed on the luminal side of the bronchial and bronchiolar epithelium in all dogs (Fig. 6). Staining of the epithelium in the terminal and respiratory bronchioles and alveolar ducts was more variable amongst dogs. In most cases, the staining was weak at the level of the terminal and respiratory bronchioles, with no visible staining towards the alveolar ducts. The columnar respiratory epithelium showed an asymmetrical staining pattern, as described for the dislodged respiratory epithelium (Fig. 6). The cuboidal epithelium of the respiratory and terminal bronchioles was more evenly stained throughout the cytoplasm and with a weaker intensity. Also, the bronchial seromucous glands showed asymmetrical cytoplasmatic staining in all dogs, with a stronger staining intensity towards the luminal side.

## Discussion

To further improve OS treatment strategies and outcomes, it is essential to understand the pathogenesis of metastasis. In this hypothesis-generating study, we have shown that it is feasible to identify pulmonary micrometastases in dogs with OS using IHC with the monoclonal antibody TP-3. All cases of canine OS previously examined with TP-3 have shown positive staining of the primary tumour [40, 41]. In the present study, we found that pulmonary metastases, microscopic metastases and micrometastases also bind TP-3. We found pulmonary micrometastases and microscopic metastases in only 20% of the dogs with OS before macroscopic metastases had developed. This was less than expected, seeing as most dogs with OS (> 90%) eventually develop pulmonary metastases, despite few having detectable metastases at presentation (< 15–17%) [31, 34, 35, 42].

One of the main concerns when using morphological methods such as IHC to detect micrometastases is that only a small portion of the entire lung can be examined. We found that micrometastases were present in three (43%) and four (57%) of the seven lung lobes examined. This finding seemingly indicates that the micrometastatic burden is relatively high once micrometastases have developed. However, our protocol might not be sensitive enough to identify dogs with a lower micrometastatic burden. It was not the scope of this study to examine the entire lung to find the true prevalence or distribution of micrometastasis. This would require the use of other methods of investigation. Without this information, we cannot evaluate the location from where tissue preferably should be sampled, nor the number of samples needed to reliably classify dogs as having micrometastases or not. Another possible explanation for the low incidence of pulmonary micrometastases could be that the lungs are not the primary site for metastatic dissemination during the early phases of metastasis in dogs with OS.

Among human patients with OS, the prevalence of tumour cells in the bone marrow at presentation was 63% and seemingly correlated with outcome [14, 43]. This is substantially higher than the prevalence of pulmonary micrometastases reported here in OS dogs. A possible explanation could be that the bone marrow serves as a temporary nest for metastasized cells until the subsequent dissemination of tumour cells to the lungs. Indeed, it has been shown that tumour cells of prostate and breast cancer in humans can disseminate to the bone marrow using mechanisms similar to those used by homing hematopoietic stem cells [44–47]. It has been suggested that these tumour cells can lay dormant in the bone marrow niche, where they remain quiescent for several years until metastases develop [48, 49]. Further studies investigating the presence of micrometastases in other organs in dogs with OS, such as the bone marrow, are therefore warranted.

We found no micrometastases or microscopic metastases in the two dogs in the OS + /Met + group (Table 2). Samples were taken from the same anatomical locations of the lungs as from the other dogs in the study, but specifically from tissues without macroscopic metastases. However, both dogs had a low metastatic burden (a total of 2 and 7 macroscopic metastases), which could indicate a less aggressive tumour phenotype. This might seem counterintuitive, as we might expect a higher micrometastatic burden once macroscopic metastases are present. However, the inhibitory effect of primary tumours on the growth of metastases is not an unknown phenomenon [50–52]. Whether overt lung metastases could exhibit a similar inhibitory effect on micrometastatic development is unknown, and further studies are needed to shed light on this. In a study evaluating the prevalence of micrometastases in seemingly unaffected areas of the lung in humans with primary lung cancer, the authors found that 47% had micrometastases [19]. Although they investigated a different cancer disease, they used a morphological method as well. They found that only 7.7% of the examined microscopic slides contained micrometastases, emphasizing the need to evaluate several samples per patient. Similarly, the prevalence of hepatic micrometastases was investigated at metastasectomy in humans with ileal, small intestinal and pancreatic neuroendocrine tumours and colorectal cancer [20–22]. The authors found micrometastases in 100, 67, 32 and 56% of patients, respectively, in seemingly unaffected liver tissue. It appears that the presence of macroscopic metastases is no guaranty for finding micrometastases when using morphological methods to detect them.

TP-3 has not been commercially available since its discovery, and there is a limited number of studies that have used the antibody. In the veterinary field, there are only two [40, 41]. In contrast to the membrane staining seen in humans, we found that TP-3 staining of tumour cells also had a cytoplasmatic distribution [38]. The previously reported staining of the brush border of ciliated epithelial cells in the bronchi and lungs of dogs, not seen in humans, corresponded well with our observations [40]. The TP-3 staining of the seromucous glands has not been described previously. This lack of specificity was not an issue when detecting micrometastases and microscopic metastases. They were easily distinguishable from normal structures based on location, clustering of cells, nuclear and cytoplasmatic morphology and staining intensity.

The number of TP-3 positive single cells varied considerably between dogs. In one case, TP-3 staining of the epithelium was present from the bronchi to the alveolar ducts, resulting in high numbers of TP-3 positive single cells per 10 HPF (case 8). New sections from this dog were sliced and stained and with the same results. This staining variation, combined with the poor morphology and dislodgement of epithelium associated with frozen sections, makes TP-3 a poor IHC marker for pulmonary single-cell metastases in dogs with OS. In most cases, distinguishing single tumour cells from dislodged respiratory epithelial cells based on staining pattern and morphology was impossible. Formalin-fixed tissue would have offered better morphology, but formalin ablates the epitope of the TP-3 [38]. We have made several attempts to optimise a protocol for formalin-fixed paraffin embedded canine tissue, but with disappointing results. Most samples were chosen from the peripheral areas of the lung (6/7 samples) to reduce the amount of respiratory epithelium included. Since TP-3 stained the respiratory epithelium down to the bronchioles and sometimes into the smaller airways, this probably had no impact on the number of TP-3 staining non-tumour cells. There was no tendency towards higher numbers of TP-3 positive single cells in the OS + groups. However, the number of dogs included is too small to draw any definite conclusions, and other methods should be considered when studying single-cell metastases.

Another limitation of our study is that the primary tumour was not IHC evaluated with TP-3. It is thus possible that some of the OS cases were TP-3 negative, and consequently, so would the micrometastases and microscopic metastases. Hence, this may have resulted in an underestimation of the micrometastatic prevalence. However, in the previous report investigating canine OS using TP-3, 13/13 tumours were TP-3 positive [40]. Likewise, in a study evaluating the use of TP-3 Fab fragments for positron-emitting tomography imaging in dogs with OS, binding was documented in the primary tumour in the four cases included [41]. In humans, 15/15 and 31/31 cases of OS examined using IHC showed strong TP-3 staining [38, 39]. There were no suspected micrometastases or microscopic metastases which did not stain with TP-3 among our dogs after careful microscopic examination of all samples.

Historically, micrometastases have been defined in different ways depending on the method of investigation and organ. When using morphological methods to identify micrometastases in lymph nodes (histology or IHC), they are generally defined as tumour cell clusters of > 0.2 mm but < 2 mm in diameter [13, 53, 54]. Clusters < 0.2 mm are usually classified as isolated (disseminated or circulating) tumour cells. Since there is no established definition for pulmonary micrometastases, we chose to define them as clusters of ≥ 5 and ≤ 50 tumour cells. Because IHC is a more sensitive method than H&E staining to identify micrometastases, we chose an upper limit of 50 cells. Clusters of > 50 cells are more easily detectable in H&E stained lung tissue, and we classified those as microscopic metastases.

Metastasis has been extensively studied in laboratory animals and in vitro models. The limited translational value of these models when developing new therapeutics has inspired researchers to create new and more sophisticated cancer models [7, 55]. Dogs with cancer have proven to be reliable clinically relevant models for human cancer, also when developing new therapeutics [56]. Given the seemingly low prevalence of pulmonary micrometastases and the fact that most eventually develop metastases, dogs with OS should serve as excellent spontaneous cancer models to study premetastatic niche formation [57]. This might include studying immunological, metabolic, and extracellular matrix changes in the lungs before metastasis has occurred. Rigorous prospective study designs and relevant controls are needed to accomplish this. If findings from murine models can be verified in a naturally occurring spontaneous cancer model such as the dog, it would further support the underlying mechanisms of premetastatic niche formation observed in mice. Since many owners decline cancer-specific treatment, a significant proportion of dogs are euthanized at an early disease stage. These dogs are excellent candidates to study distant metastatic target organs early in the disease process, to an extent not feasible in humans. Furthermore, by verifying findings from murine models, dogs could serve as a bridge between the preclinical models and humans for developing new therapies targeting premetastatic niche formation. We propose that dogs with spontaneous appendicular OS could represent clinically relevant models for studying early micrometastasis and premetastatic niche formation as an addition to murine models.

## Conclusions

Our data shows that pulmonary micrometastases can be detected in dogs with OS by using TP-3 immunohistochemistry. The prevalence of pulmonary micrometastases was significantly lower than expected in dogs with OS before macroscopic metastases had developed. Once present, the micrometastatic burden was relatively high. This could indicate that pulmonary metastases do not originate directly from the primary tumour. However, it remains a hypothesis-generating study, and larger studies are needed to validate our findings.

## Data Availability

The data that support the findings of this study are available from the corresponding author upon reasonable request.
